# Improving cancer clinical trial enrollment and patient understanding using TrialTalk, a structured communication method: a pilot randomized controlled trial

**DOI:** 10.1007/s00520-026-10505-4

**Published:** 2026-03-24

**Authors:** Toby C. Campbell, Cassandra Gedeon, Bonyan Qudah, Mallory Jasicki, Kristine L. Kwekkeboom, Amy Zelenski

**Affiliations:** 1 School of Medicine and Public Health, University of Wisconsin-Madison, Madison, WI 53705 USA; 2 LAPSCO UMR6024, University Clermont Auvergne, Clermont-Ferrand, France; 3 School of Nursing, University of Wisconsin-Madison, Madison, WI 53705 USA

**Keywords:** Clinical trials, Decision-making, Patient–provider communication, Decision tools, TrialTalk

## Abstract

**Introduction:**

Structured, easy-to-interpret approaches are needed to facilitate preference-sensitive decision-making about cancer treatments. The TrialTalk method incorporates a verbal component and a pen-and-paper diagram that outlines the diagnosis, prognostic implications, treatment options, potential outcomes, and anticipated impacts on daily life. This pilot study examined (1) oncologists’ ability to learn and then implement the tool in their clinical practice and (2) the effect of the TrialTalk method on clinical trial consent and enrollment.

**Methods:**

Twenty-seven oncologists from a single academic institution were randomly assigned to the intervention group (*n* = 14) or the control group (*n* = 13). Intervention group oncologists completed a single, 2-hour TrialTalk training program including a didactic, simulated session with patient actors and feedback from the trainer. Additional feedback and question/answer sessions were available. Oncologists in the control group did not receive TrialTalk training. Clinical trial decisions were collected from patients seen by oncologists in both groups.

**Results:**

Intervention oncologists demonstrated fidelity with the tool after training. Patients of oncologists in the intervention group were significantly more likely to consent to participate in clinical trials than patients of oncologists in the control group (92.9% vs. 82.4%, *p* = 0.04). Actual enrollment rates after signing consent were equal in both groups (78.2% in the intervention group vs. 73.3% in the control group).

**Conclusion:**

Patients who met with TrialTalk-trained oncologists were more likely to sign consent to participate in a clinical trial. The decision-making conversation with the oncologist is a critical moment for patients considering clinical trial participation, and targeting these encounters has the potential to increase overall trial participation rates.

**Trial registration:**

ClinicalTrials.gov ID: NCT03656276.

## Introduction

Critically low participation rates in clinical trials prolong the development of new treatment options and disease understanding for people with cancer, prompting the question: What more could be learned if trials met their enrollment targets faster? Estimates indicate that only 7.1% of adult cancer patients participate in clinical trials, a barrier that impedes our potential for progress in cancer treatment [1]. While numerous factors contribute to study participation rates, the quality of patient-clinician decision-making conversations is a crucial, modifiable factor that can increase participation by ensuring that all potentially interested patients are identified and offered the opportunity to participate [2, 3].

Two significant patient-related barriers to clinical trial enrollment are low health literacy and a limited understanding of clinical trials [4, 5]. Many patients find it challenging to comprehend complex information about trial participation, including the nature of trials, potential risks and benefits, and the significance of participation for them as unique individuals [5, 6]. At their worst, these conversations can breed distrust, block patients from considering a trial, and damage their relationship with their oncologist. As a result, these conversations are high risk for both oncologists and patients.

By optimizing the physicians’ approach to this important decision-making conversation, physicians can help patients make goal-concordant decisions about treatments while maintaining a positive relationship. Physicians can optimize these conversations by explaining cancer treatment options clearly and comprehensively, while addressing any concerns and misconceptions that patients may have. Highlighting the personal and altruistic benefits of participating in clinical trials can motivate patients who would be willing to contribute to medical research while also potentially gaining access to new experimental therapies [7]. Tailoring the information to the individual can make it more relevant and engaging, thus enhancing the patient’s understanding and willingness to participate [8, 9].

In response to this challenge, we developed TrialTalk, a structured communication method designed to enhance the clarity and effectiveness of treatment decision-making conversations. In this manuscript, we introduce the TrialTalk method and present the results of a pilot randomized trial conducted in a single academic institution. Preliminary findings of this study will be used to support the implementation and testing of TrialTalk in a larger, multisite study and optimize data collection methods used to evaluate the adoption and fidelity of the TrialTalk method.

### The TrialTalk method

The TrialTalk method is situated within Elwyn’s (2010) conceptual model of shared decision-making and has foundations in cognitive science regarding how humans make decisions [10]. TrialTalk development was informed by the extensive work done in our lab to analyze and describe routine clinical discussions in oncology [11–14]. TrialTalk provides a structure for the information typically covered by oncologists. It has two components: verbal conversation and a companion pen-and-paper diagram created before or during the conversation. With TrialTalk, oncologists verbally cover six core elements: (1) explaining diagnosis and prognostic implications, (2) indicating to the patient that they have multiple options and a decision to make, (3) using narrative to describe the lived experience of each one of the three options (a reduced intensity option such as surveillance or best supportive care alone, standard of care option, and clinical trial option), (4) comparing options using the same efficacy outcome (e.g., 2-year survival rate), (5) inviting deliberation, and (6) making a recommendation that aligns with patients’ preferences [15]. Concurrently, a simple diagram is constructed with pen and paper, which summarizes the six elements of the discussion. Each option is detailed with treatment name, potential side effects, and impact on the patient’s life [16].

**Fig. 1:**
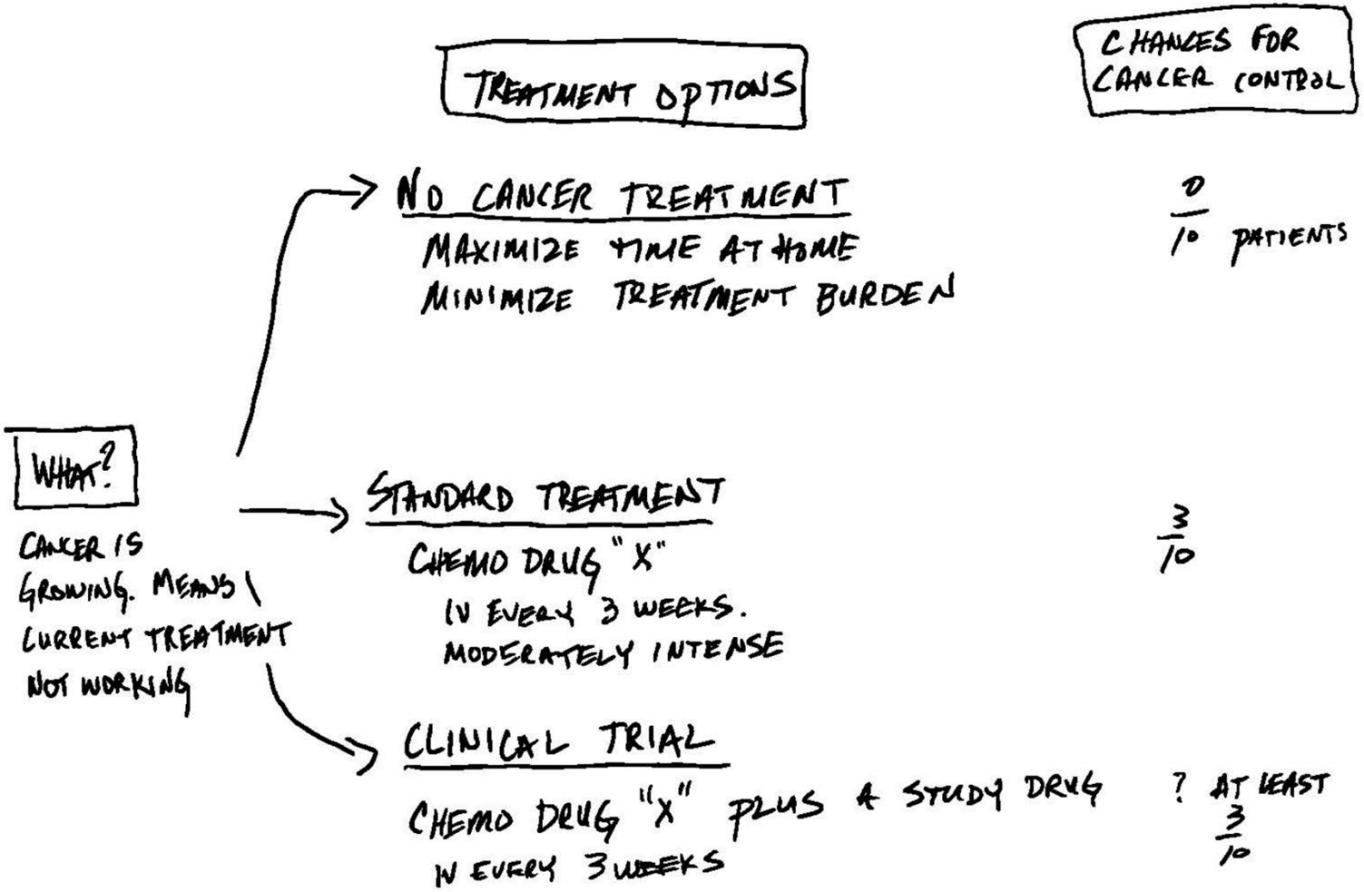
Sample TrialTalk diagram for a patient with progressive disease on current therapy who has a phase 2 clinical trial option

Providing take-home aids enhances patients’ memory and facilitates high-quality deliberation with their support network after the visit (Fig. 1) [9, 17, 18]. Moreover, by systematically discussing the risks, benefits, and alternatives of treatment options while documenting the patient’s preferences and choices, TrialTalk is designed to meet all legal and ethical requirements for informed consent discussions as outlined by major oncology societies such as the American Society of Clinical Oncology [19].

### Study objectives

The primary objectives of this study are the following:

1. Evaluate the impact of TrialTalk training on oncologists’ competency in using the TrialTalk method.
2. Examine the impact of TrialTalk method on clinical trial outcomes, including referral to the clinical trial coordinator, signed consent, and enrollment in clinical trials, compared to usual care.

## Method

### Design

This was a pilot randomized controlled trial to evaluate training and the preliminary effects of TrialTalk at an academic medical center in Wisconsin. Oncologists were randomized into an intervention group (TrialTalk communication training) or a control group (usual care without communication training). Randomization was done using computer-generated random numbers to assign providers to either group with an allocation ratio of 1:1. The study adhered to the CONSORT guidelines for reporting parallel randomized clinical trials [20].

### Participants

Oncologists were invited to participate through division and research group meetings. The staff lists from different divisions were also used to identify potential oncologists. To be eligible, oncologists had to engage with patients about treatment decisions, including therapeutic clinical trials. Any oncologist who had enough knowledge of the TrialTalk communication intervention to render the randomization process ineffective was excluded from participation. Additionally, oncologists with low patient volume (who see fewer than four patients/week in clinic) were excluded.

Research staff scheduled face-to-face meetings with interested providers to share more study information. Participants completed the informed consent process during the meeting, and all participants provided written consent to participate in the study.

Patients seen by enrolled oncologists were included in the analysis if they were 18 years of age or older, able to read and understand English, and participated in a discussion about treatment options with their provider, including a therapeutic clinical trial. Patients were excluded if they were blind or deaf, had no capacity for decision-making, or were offered a phase 1 clinical trial. The latter were excluded due to the non-therapeutic intent of phase 1 trials.

### Procedure

The University of Wisconsin Minimal Risk Research Institutional Review Board approved the study protocol (UW16060). The clinical trial was conducted in accordance with the ethical principles that have their origin in the Declaration of Helsinki and that are consistent with good clinical practice (GCP).

### Funding declaration

This study was funded by the Carbone Cancer Center and the UW Institute for Clinical and Translational Research.

### TrialTalk training

We conducted TrialTalk training sessions in a conference room. Each oncologist in the intervention group participated in two 1-hour sessions. During the first session, a trainer presented background information on TrialTalk, taught the verbal approach and how to create the pen-and-paper diagram, and oncologists practiced constructing diagrams using a sample case. In the second session, we covered skills training, which included creating TrialTalk diagrams and receiving individualized feedback from the instructor, role-plays to practice the verbal aspects of TrialTalk with a patient actor, and group feedback from peers and the facilitator. Patient actors were hired from the University of Wisconsin School of Medicine and Public Health.

Following training, participants conducted a treatment decision-making visit with a standardized patient and were evaluated by the trainer using a fidelity checklist to assess whether oncologists completed the essential components of the TrialTalk method. Oncologists who did not meet the fidelity criteria were offered follow-up face-to-face meetings with the research team and optional simulated training. These sessions helped address gaps in using the TrialTalk method and reinforced the verbal and diagrammatic skills required for effective use.

### Data collection

Once an oncologist completed training and demonstrated the required level of competence, research staff collected data about clinical trial discussions. To identify patients who had clinical trial discussions, oncologists were provided with a daily clinic schedule so they could simply check a box next to the names of patients with whom they discussed clinical trials. Then, a chart review was done to examine whether patients scheduled a meeting with a trial coordinator and their consent and actual enrollment in clinical trials. The demographics of included patients were also collected from the medical records. Clinical trial data were collected from included patients until they received their first dose of therapy (clinical trial, standard therapy, or no therapy). To track whether intervention oncologists discussed the clinical trial option using TrialTalk, clinical staff collected TrialTalk diagrams from the oncologist or patient and scanned and uploaded them into the electronic medical records.

### Sample size calculation

The primary outcome was the clinical trial recruitment rate (patient signs consent). We anticipated that the proposed intervention would increase the recruitment rate from 30% to 40–50%. To achieve 80% power at a one-sided 0.05 significance level with 1:1 randomization, the study required at least 50 oncologists (25 per group), each enrolling an average of 15 patients.

### Outcome measures

*Competence in the TrialTalk method*: The effectiveness of the TrialTalk training was assessed using a fidelity checklist. The checklist encompassed the verbal and diagrammatic elements. Four coders received training on scoring diagrams by open-coding four diagram samples until a consensus was reached and then coded the rest of the diagrams independently. Acceptable use of the TrialTalk was determined by a score of 14 points or higher out of a possible 20. *Clinical trial decisions:* We recorded the number of patients approached with a clinical trial option from enrolled oncologists’ daily clinic checklists. We used the electronic medical records and institutional clinical trial database to track the number of patients who agreed to meet with a clinical trial coordinator (referral rate), the number of patients who signed consents (recruitment rate), and the number of patients who were found eligible and began therapy on the trial (enrollment rate).

### Data analysis

All statistical tests were run using R Studio (version 2022), and the significance level was set at 0.05. Characteristics of the study sample were summarized using standard descriptive statistics stratified by the study arm. The association between TrialTalk use and clinical trial decisions was assessed using the chi-square method. We used a generalized linear mixed-effects model (GLMM) with a logit link function and oncologist-specific random effects to compare referral rates between study groups. Additionally, a fixed-effects generalized linear model (GLM) was employed to assess the likelihood of signing consent among participants who met with the trial coordinator, adjusting for patient demographics. Finally, we ran an exploratory subgroup analysis to assess the differences between study groups in consenting to clinical trials based on disease and trial type using the chi-square method.

## Results

### Baseline data

**Fig. 2:**
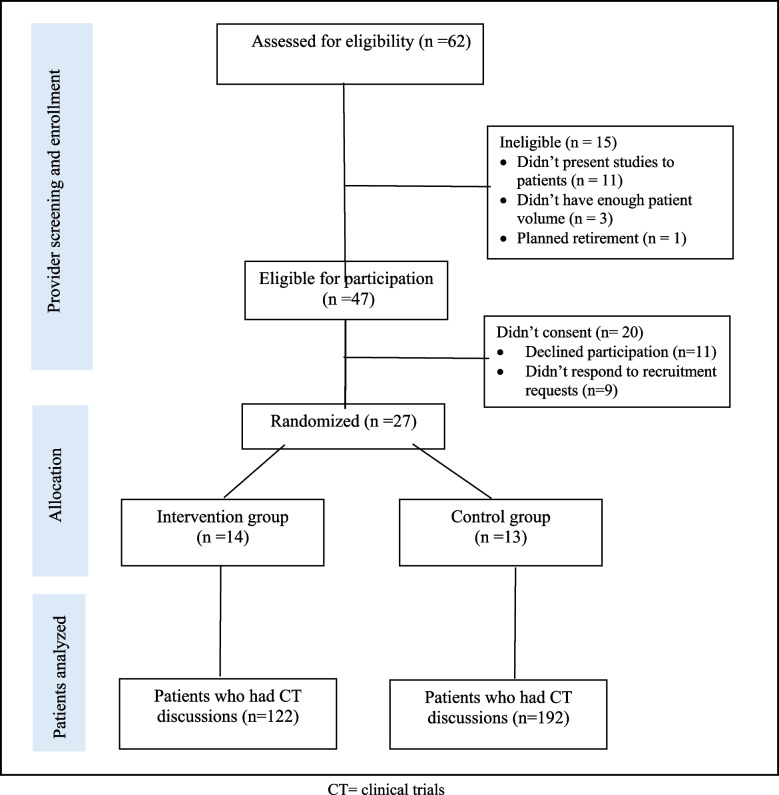
Study flowchart. CT = clinical trials

Participant recruitment and data collection began in March 2017 and ended in March 2019. The study flow is shown in Fig. 2.

#### Oncologists

Twenty-seven oncologists participated in the study, and they had comparable demographic characteristics, as shown in Table 1. The 14 oncologists in the intervention group received TrialTalk training, and 13 oncologists were in the usual care group.

**Table 1:** Oncologists’ demographics per study group

	Intervention *n* = 14		Control *n* = 13		Combined *N* = 27	
	*N*	%	*N*	%	*N*	%
Gender						
Men	6	43	8	62	14	52
Women	8	57	5	38	13	48
Subspecialty practice						
Breast	2	14	1	8	3	11
Gastrointestinal	2	14	2	15	4	15
Urological	1	7	1	8	2	7
Gynecology	1	7	2	15	3	11
Hematologic	2	14	1	8	3	11
Lymphoma/myeloma	1	7	2	15	3	11
Radiation	2	14	1	8	3	11
Thoracic	1	7	2	15	3	11
General oncology	2	14	1	8	3	11
Completed initial training	14	100	0	0	14	52
Second training	2	14	0	0	2	7
Follow-up sessions	2	14	0	0	2	7
	Median	Range	Median	Range	Median	Range
Number of patients/oncologist	9	2–17	9	1–38	9	1–38

#### Patients

Three hundred and fourteen patients (*M*_age_ = 61.1 years; *SD*_age_ = 11.38 years) had clinical trial discussions with 27 oncologists. Their demographic information is displayed in Table 2. Out of the total patients, 122 were in the intervention condition, and 192 were in the control condition. Twenty-one percent of the patients (*n*= 66) had previously participated in a clinical trial before the current conversation. Interestingly, the data show that providers in the intervention group discussed clinical trials at a significantly higher rate with female patients compared to males (75% vs. 25%). Sub-analysis of the characteristics of the intervention group reveals that over half of clinical trial discussions (52.5%) were conducted with patients diagnosed with gynecologic cancer (breast, uterine, and ovarian cancers). As studies show that women often seek greater involvement in shared-decision-making consultations and more comprehensive information about their treatment options, providers likely used TrialTalk as a direct response to meet their needs [21, 22].

**Table 2:** Patients’ demographics per study group

	Intervention *n* = 122		Control *n* = 192		Combined *N* = 314	
	*N*	%	*N*	%	*N*	%
Gender						
Male	31	25	106	55	137	44
Female	91	75	86	45	177	56
Race						
White	120	98	182	95	302	96
Black/African American	1	0.8	6	3.00	7	2
Asian	0	0	1	0.5	1	0.3
American Indian/Alaska Native	1	0.8	0	0	1	0.3
Native Hawaiian or Pacific Islander	0	0	1	0.5	1	0.3
Other	0	0	2	1	2	0.6
Ethnicity						
Hispanic	2	2	10	5	12	4
Non-Hispanic	120	98	182	95	302	96
Previous clinical trial	33	27	33	17	74	21
Cancer stage*						
Local	8	7	16	8	24	8
Regional	42	34	51	27	93	30
Advanced	40	33	103	54	143	46
Insufficient staging data	18	15	16	8	34	11
	Median	Range	Median	Range	Median	Range
Age (years)	59	28–87	63	29–86	62	28–87

*14 missing data for the intervention group, 6 missing for the control group

### Training outcomes

A total of three training workshops were conducted with oncologists, which were facilitated by the TrialTalk designer. The average fidelity score among participants was 14.8 ± 2, with scores ranging from 10 to 18. Twelve out of the 14 oncologists in the intervention group demonstrated competence in using the communication method after 2 hour of training. Two oncologists had follow-up meetings with the research team and additional simulated training to improve their skills in using the TrialTalk method, leading to increased fidelity scores.

### TrialTalk use and clinical trial decision outcomes

Obtaining completed diagrams from intervention patients was challenging. Diagrams were only obtained from 45% (55/122) of oncologists in the intervention group. Thus, we were not able to assess if TrialTalk was consistently used with each patient in the intervention group.

A significant difference was observed in the referral rate of patients to the clinical trial coordinator, with higher proportions of patients in the control group who accepted the referral (Table 3). However, when we fitted the data in a GLMM to account for the random effects of oncologists, there was no significant difference between groups (*β* = −1.04, 95% CI [0.55, −1.9], *p* = 0.06).

**Table 3:** Clinical trial decisions per study group

	Intervention group (*n* = 122)	Control group (*n* = 192)	*p *value
Accepted referral to the trial coordinator	71.7% (86/120)^α^	83.3% (160/192)	0.02
Consent signed^a^	92.9% (78/84)^α^	82.4% (131/159)^β^	0.04
Clinical trial enrollment^b^	78.2% (61/78)	73.3% (96/131)	0.53

^α^Two missing data

^β^One missing data

^a^Among patients who agreed to talk with a clinical trial coordinator

^b^Among patients who signed the consent

Among those who met with the trial coordinator, patients in the intervention group were more likely to sign the consent form for clinical trial participation (92.9%) compared to those in the control group (82.4%), *Χ*^2^ (1, 243) = 4.17, *p* = 0.04. Lastly, there was no significant difference between study groups in clinical trial enrollment among patients who signed the consent form, *Χ*^*2*^ (1, 209) = 0.40, *p* = 0.53. This indicates that, once the consent was signed, patients were equally likely to enroll in the clinical trial regardless of the initial communication strategy.

Another analysis was conducted to estimate the odds of signing consent among study groups while controlling for patients’ demographics (age and gender). Findings show that patients in the control group had 67% lower odds of signing a consent compared to patients in the intervention group (OR = 0.33, 95% CI [0.12, 0.80], *p* = 0.02), which was not influenced by age or gender.

The impact of using the TrialTalk method did not differ between study groups based on the cancer diagnosis. For example, in the breast cancer group, 91% of patients in the intervention group signed the consent form, compared to 76% in the control group (*p* = 0.24). Similar patterns were observed across other cancer diagnoses, including gastrointestinal, genitourinary, gynecologic, hematologic, and thoracic.

Moreover, we examined the method’s impact across different types of clinical trials. For randomized trials without a placebo, the intervention group had a significantly higher consent rate (94% vs. 75%, *p* = 0.02). For randomized trials with a placebo and non-randomized trials, consent rates were similar between groups.

## Discussion

The results of our study indicate that the TrialTalk method can effectively improve oncologists’ ability to present treatment options to patients, ultimately enhancing patients’ willingness to participate in clinical trials. The significant association between the intervention and the likelihood of signing consent to participate underscores the potential of a structured communication framework to bridge gaps in understanding and address barriers to clinical trial participation. By providing a structured approach and creating individualized educational materials, TrialTalk is believed to demystify clinical trials and foster a more informed and engaged patient population [23]. This is particularly important given the complex and often overwhelming nature of clinical trial information [5]. However, it is important to note that this was a pilot randomized controlled study searching for evidence about the potential of a structured communication method to facilitate the discussion between patients and providers about cancer clinical trials. Due to the limited sample size and the restriction to one healthcare institution, the results should be interpreted with caution. Several important findings will be discussed here that have implications for implementing and evaluating TrialTalk’s impact in future studies.

Our findings indicate that TrialTalk primarily impacted barriers downstream of the referral process, such as improving patient education or addressing fear or trust issues, as suggested by the lack of impact on referral rate. Underlying differences among oncologists in communication style or referral practices might have affected referral rates. Additionally, TrialTalk likely served as a prescreening tool, ensuring that only patients who developed a well-rounded understanding and a genuine interest in clinical trials would proceed to meet with the trial coordinator.

With regard to actual clinical trial enrollment, a higher percentage of intervention patients ultimately enrolled in clinical trials compared to those in the control group. However, this difference did not reach significance. Protocol-related barriers or health deterioration might have influenced patients’ decisions to enter clinical trials, which warrants exploration in future studies [24].

The observed higher consent rate to randomized controlled trials with no placebo by patients in the intervention group is worth further exploration, as our study was not designed to answer this question. A possible explanation for this difference is that patients in the control group did not fully understand the benefits of these kinds of trials compared to other types or had doubts about trial participation that went unaddressed.

The implications of our study are multifaceted. First, the study highlights that even brief, targeted training can significantly enhance an oncologist’s ability to communicate complex information effectively. This finding supports the incorporation of communication training into oncology education and professional development programs to improve cancer care delivery [25]. Second, given the low participation rates in clinical trials, implementing the TrialTalk method could lead to more representative trial populations, ultimately accelerating the development of new cancer treatments. Though it was not explored in this study, TrialTalk has the potential to reduce disparities in doctor–patient communication, particularly among marginalized racial groups. Research has shown that communication barriers are often more pronounced in interactions with patients from racial and ethnic minorities [26, 27]. By standardizing the communication process, TrialTalk can ensure that all patients receive clear and consistent information, thereby promoting equity in medical interactions [9]. An implementation study is underway to explore TrialTalk’s impact in promoting equitable clinical trial accrual among Black and white patients (ClinicalTrials.gov ID NCT06985953).

While the initial results are promising, several limitations must be acknowledged. Firstly, the study was conducted at a single institution, which may limit the generalizability of the findings. Recruiting oncologists was challenging due to providers’ busyness. The small sample size might have reduced the power to identify significant differences in several primary and secondary outcomes. Additionally, all doctors were trained by TrialTalk’s designer, which might have influenced the outcomes. We also relied on oncologists’ self-reports to identify which patients had a trial presented to them, introducing potential bias. It was also unclear whether TrialTalk was consistently used with all intervention patients who participated in clinical trial discussions, as obtaining diagrams from all intervention oncologists was not feasible. This suggests that different methods should be devised to measure adoption and fidelity in practice to ensure that the observed differences in clinical trial decisions are specifically related to TrialTalk use. It was noted that clinical trials were discussed at a higher rate by providers in the control group, which can be explained by the “John Henry Effect,” where participants in the control group feel disadvantaged and work extra hard to outperform those in the intervention group [28]. Cross-group contamination and the control group’s awareness of the intervention might have taken place and contributed to this effect. Finally, further research is needed to explore the method’s long-term impact on patient outcomes.

## Conclusion

In conclusion, findings from this pilot study suggest that the TrialTalk method can potentially advance patient-centered care and clinical trial recruitment. By enhancing the clarity and quality of physician–patient communication, TrialTalk addresses a critical gap in the current oncological landscape. Larger, multisite studies are urgently needed to build on these findings to optimize communication strategies and support informed and preference-sensitive decision-making in cancer care. Additionally, exploring effective implementation and dissemination methods for TrialTalk is essential to ensure its accessibility and sustainability.

## Data Availability

No datasets were generated or analysed during the current study.
